# Comparative genomics of the primary endosymbiont *Buchnera aphidicola* in aphid hosts and their coevolutionary relationships

**DOI:** 10.1186/s12915-024-01934-w

**Published:** 2024-06-20

**Authors:** Yukang Liang, Rebecca B. Dikow, Xu Su, Jun Wen, Zhumei Ren

**Affiliations:** 1 School of Life Science and Shanxi Key Laboratory of Nucleic Acid Biopesticides, Shanxi University, 92 Wucheng Rd, Taiyuan Shanxi, 030006 China; 2 Data Science Lab, Office of the Chief Information Officer, Smithsonian Institution, 600 Maryland Avenue SW, Washington, DC 20024 USA; 3 School of Geography and Life Science, Qinghai Normal University, 38 Wusixi Road, Xining, 810008 China; 4 Department of Botany, National Museum of Natural History, Smithsonian Institution, MRC-166, Washington, DC 20013-7012 USA

**Keywords:** *Rhus* gall aphid, Endosymbiont, *Buchnera*, Phylogenomics, Coevolution

## Abstract

**Background:**

Coevolution between modern aphids and their primary obligate, bacterial endosymbiont, *Buchnera aphidicola*, has been previously reported at different classification levels based on molecular phylogenetic analyses. However, the *Buchnera* genome remains poorly understood within the *Rhus* gall aphids.

**Results:**

We assembled the complete genome of the endosymbiont *Buchnera* in 16 aphid samples, representing 13 species in all six genera of *Rhus* gall aphids by shotgun genome skimming method. We compared the newly assembled genomes with those from GenBank to comprehensively investigate patterns of coevolution between the bacteria *Buchnera* and their aphid hosts. *Buchnera* genomes were mostly collinear, and the pan-genome contained 684 genes, in which the core genome contained 256 genes with some lineages having large numbers of tandem gene duplications. There has been substantial gene-loss in each *Buchnera* lineage. We also reconstructed the phylogeny for *Buchnera* and their host aphids, respectively, using 72 complete genomes of *Buchnera*, along with the complete mitochondrial genomes and three nuclear genes of 31 corresponding host aphid accessions. The cophylogenetic test demonstrated significant coevolution between these two partner groups at individual, species, generic, and tribal levels.

**Conclusions:**

*Buchnera* exhibits very high levels of genomic sequence divergence but relative stability in gene order. The relationship between the symbionts *Buchnera* and its aphid hosts shows a significant coevolutionary pattern and supports complexity of the obligate symbiotic relationship.

**Supplementary Information:**

The online version contains supplementary material available at 10.1186/s12915-024-01934-w.

## Background

Insects are the most diverse and abundant class of animals on earth and are associated with a remarkable range of symbiotic microorganisms. Many insects are well known to have co-diversified with intracellular bacterial symbionts, or endosymbionts, including nearly all groups of phloem sap-sucking insects [1–3]. Among plant-sap-sucking insects, aphids comprise around 4000 species mainly distributed throughout the temperate regions of the globe [4] and constitute a monophyletic group, superfamily Aphidoidea, within the order Hemiptera [5]. Most aphids have an obligate, mutualistic relationship with the symbiotic bacteria, *Buchnera aphidicola* (Proteobacteria: Gammaproteobacteria: Enterobacteriaceae) inhabiting specialized cells called bacteriocytes, which occur in the aphid’s abdominal haemocoel [6–8]. *Buchnera aphidicola* is required for host development, growth, and reproduction [9, 10]. *Buchnera* provides the host aphid with nutrition such as amino acids, vitamins, and sterols, which are all necessary for normal development and reproduction but cannot be synthesized by the aphids and are deficient in their phloem sap diet [11–13]. In turn, the aphid provides *Buchnera* with nutrients, including nonessential amino acids and carbohydrates that are abundant in the phloem diet or produced by the aphid host [10, 14]. The bacteria are also completely dependent on the aphid for vertical transmission through maternal lineages [3, 11]. Recently, comparative whole-genome sequence analysis of the pea aphid, *Acyrthosiphon pisum*, and its primary endosymbiont revealed that these two obligate mutualists were fully interdependent for the biosynthesis of amino acids, and the two genomes formed a highly integrated metabolic collaboration [13, 15–17]. The relationship between *Buchnera* and their host aphids is at least 150 million years old [18, 19] and has been considered one of the best-studied cases of symbiosis and coevolution [20–24].

The term “coevolution” was first introduced in a study on butterflies and their plant hosts in 1964 [25]. Co-phylogenetic analysis of the host and parasite trees have been the main method for the study of coevolution with rapid development of molecular phylogenetic techniques [26]. The degree of congruence reflects whether parasites and their hosts have undergone co-diversification, in which one organismal partner triggers diversification in the other, or if they have random associations in their evolutionary history [27, 28]. Co-diversification may be investigated by comparing the topologies of phylogenetic trees of the host and parasite lineages. The topologies might be congruent if co-diversification has occurred, and the timing of diversification in both lineages should also be correlated in a co-diversification scenario [26, 29].

Different strains of this symbiotic bacteria *Buchnera* within different aphid hosts have been named as one species, i.e., *Buchnera aphidicola* [20]. The co-diversification between *Buchnera* and its host aphid has been investigated in prior studies at different taxonomic scales. These studies mainly used the 16S ribosomal RNA gene for phylogenetic reconstruction and focused on closely related species of aphids or intra-species lineages [21, 22, 30–33]. Recently, Arab and Lo (2021) suggested a highly significant correlation of molecular rates between the genomes of *Buchnera* and the mitochondrial genomes of their hosts [34]. These analyses usually supported parallel evolution and co-diversification in the aphid-*Buchnera* associations and suggested that the *Buchnera* gene sequences could be used as molecular markers to define the species and evolutionary relationships of the aphids [21, 30].

Since the complete genome of *Buchnera aphidicola* sp. strain APS from the pea aphid (*Acyrthosiphon pisum*) was first sequenced and analyzed [15], so far, there have been 73 complete genomes of *Buchnera* reported in GenBank from 56 aphid species (Additional file 1: Table S1), belonging to the family Aphididae and representing eight subfamilies, i.e., Aphidinae, Eriosomatinae, Lachninae, Calaphidinae, Phyllaphidinae, Hormaphidinae, Thelaxinae, and Anoeciinae [15, 19, 35–40]. In total, the *Buchnera aphidicola* genomes range from 412 to 646 kb in length, thus comprising one of the smallest known cellular genomes of symbiotic bacteria [34, 36, 38, 41–43]. *Buchnera* has experienced drastic shrinkage in genome size, retaining only essential genes for its specialized lifestyle, and genome shrinkage may be ongoing [15, 38, 44–46].

The *Buchnera* genome remains poorly understood within the *Rhus* gall aphids of the subtribe Melaphidina (Aphididae: Eriosomatinae: Fordini) [5, 47, 48]. The Melaphidina aphids live obligately on species of sumac plants (*Rhus* subgenus *Rhus*, Anacardiaceae) as the primary host for part of their life cycle to induce a large gall on a developing leaf or shoot [49]. The gall is rich in tannin and, therefore, effective in traditional medicine for stopping dysentery, bleeding, coughing, sweating, and rectal prolapse [49]. Also, tannins are extracted from the galls and further chemically synthesized as a series of ester compounds, which are widely applied as additive component in various fields, such as medicines, electronics, chemical products, oils, inks, dyeing, and foods [50]. The Melaphidina aphids, like other aphids, are believed to have an obligate, mutualistic relationship with their endosymbiotic *Buchnera aphidicola* [4, 18, 51, 52]. The genomes of *Buchnera* in Melaphidina have not been studied comprehensively, and only two genome sequences of *Buchnera* strains from Melaphidina were published [38]. Yet, little is known about the co-evolutionary patterns between *Rhus* gall aphids and their primary symbiont *Buchnera*.

In this study, we sequenced the complete genomes of *Buchnera aphidicola* in *Rhus* gall aphids and investigated the co-evolution of *Buchnera* and its host aphids within a phylogenomic framework. We obtained 15 complete genomes of *Buchnera aphidicola* in 17 samples of the 16 *Rhus* gall aphid species (including three subspecies and two individuals for the species *Nurudea yanoniella*) and *Chaetogeoica yunlongensis*, a species in the Fordina subtribe sistering to *Rhus* gall aphids. Each complete genomes of *Buchnera aphidicola* is derived from only one species or subspecies of *Rhus* gall aphids, including all six genera and the 13 species recognized within Melaphidina. We combined these newly generated genomes with the existing molecular sequences for *B. aphidicola* in other aphids from GenBank to construct the *Buchnera* phylogeny, along with the aphid phylogeny based on complete mitochondrial genome and three nuclear genes to investigate coevolution and/or co-diversification of these endosymbionts and their host aphids, while we especially focused on *Rhus* gall aphids and their symbiont *Buchnera*. Our work aims to yield new insights into biodiversity of *Buchnera* and into the coevolutionary patterns of the aphid-bacterial system.

## Results

### *Buchnera* genomics and phylogenomics

All the newly sequenced bacterial genomes are available from the SRA database of NCBI as raw data, from that we assembled and annotated genomes for 15 newly sequenced *Buchnera* samples, which were deposited and available in GenBank (Table 1). For seven of the 15 genomes, we were able to assemble a single circular chromosome with one contig and other nine with more than one contig (Table 1). There were two *Buchnera* samples in *Kaburagia rhusicola ovatirhusicola* and *K. r. ovogallis* strain for which we did not recover sequences of *Buchnera* even though these species are assumed to contain the symbionts (Codes R9 and R11 in Table 1). These aphids and bacterial symbionts will be subject in future investigation to determine whether these results are due to poor bacterial preservation or unexpected biological loss of the association.

**Table 1:** Information of aphid genes from GenBank (also our previous study) [53–55] and its symbiont *Buchnera* genome obtained in this study

Code	Species	Voucher#	# of bases	GenBank accession no				Symbiont *Buchnera*						
				Mt	EF1-α	LWO	18S	Code	Genome size (bp)	# CDs	GC%	# of contigs	BioProject #	Accession No
R1	*Schlechtendalia chinensis*	A1798	6.1G	KX852297	KF601635	MF179857	-	BSc	607,700	544	25.8	1	PRJNA1025172	CP140041
R2	*Schlechtendalia peitan*	A242	5.9G	MF043979	MF159563	MF179858	MF152695	BSp	625,015	550	24.9	2	PRJNA1025172	CP140043
R4	*Floraphis choui*	A403	7.1G	MF043980	MF152697	MF179853	MF152688	BFc	616,219	540	24.6	1	PRJNA1025172	CP140044
R6	*Meitanaphis flavogallis*	A2012	5.1G	MF043982	MK424022	MK412096	MF280270	BMf	619,106	533	25.5	2	PRJNA1025172	CP140038
R7	*Kaburagia rhusicola ensigallis*	A1126	5.8G	MF043984	MF152699	MF179859	MF152690	BKre	617,138	534	26.0	1	PRJNA1025172	CP140040
R8	*Meitanaphis elongallis*	A250	5.2G	MF043989	MF152700	MF179855	MF152692	BMe	625,615	540	26.0	1	PRJNA1025172	CP140033
R9	*Kaburagia rhusicola ovatirhusicola*	A1513	5.1G	MF043985	MK424019	MK412094	MF280268	BKro	X	X	-	X		
R11	*Kaburagia rhusicola ovogallis*	A174	5.1G	MF043986	MF159561	MF179860	MF152691	BKro	X	X	-	X		
R12	*Kaburagia rhusicola rhusicola*	A1539	4.7G	MF043987	MK424021	MK412095	MF280269	BKrr	617,062	540	26.0	2	PRJNA1025172	CP140032
R13	*Nurudea yanoniella*	A1677	4.2G	MK435595	MF152701	MF179856	MF152694	BNy	608,857	540	24.2	4	PRJNA1025172	CP140036
R14	*Nurudea yanoniella*	A267	3.5G	MF043983	MK424024	MK412098	MF280273	BNy	604,433	532	24.3	9	PRJNA1025172	CP140034
R15	*Nurudea ibofushi*	A1796	5.1G	MF043981	MK424020	MK412097	MF280271	BNi	606,188	544	25.8	7	PRJNA1025172	CP140039
R17	*Chaetogeoica yunlongensis*	A313	3.5G	MF043988	MF152696	-	MF152687	BBy	588,399	506	23.6	2	PRJNA1025172	CP139909
R20	*Nurudea shiraii*	A184	3.9G	MF043978	MF152701	MF179856	MF152694	BNs	607,770	532	24.9	1	PRJNA1025172	CP140035
R22	*Melaphis rhois*	A3037	4.0G	KY624581	MF159562	-	MF152693	BMr	623,623	549	25.6	1	PRJNA1025172	CP140031
R23	*Floraphis meitanensis*	A118	4.7G	MF043990	MF152698	MF179854	MF152689	BFm	616,236	539	24.6	2	PRJNA1025172	CP140037
R24	*Meitanaphis microgallis*	A4540	6.5G	MK948431	OR611940	OR611939	OR676345	BMm	626,129	543	25.9	4	PRJNA1025172	CP140042

We downloaded 57 genomes of *Buchnera* in other aphids from GenBank (Additional file 1: Table S1) as well as our current *Buchnera* genomes to compare and analyze their general characters. As a result, we found that the 72 *Buchnera* genomes ranged from 412,404 to 645,850 bp in length with a strong bias towards A + T (71.9 to 79.9%). All the symbiont genomes in *Rhus* gall aphids ranged from 604,433 to 626,129 bp and contained 532 to 550 protein-coding genes, 31 to 33 tRNAs, one ncRNA, one tmRNA, and three rRNAs. These genomes of *Buchnera* in different aphid species are relatively stable in terms of gene order, in contrast to the high levels of nucleotide divergence.

**Fig. 1:**
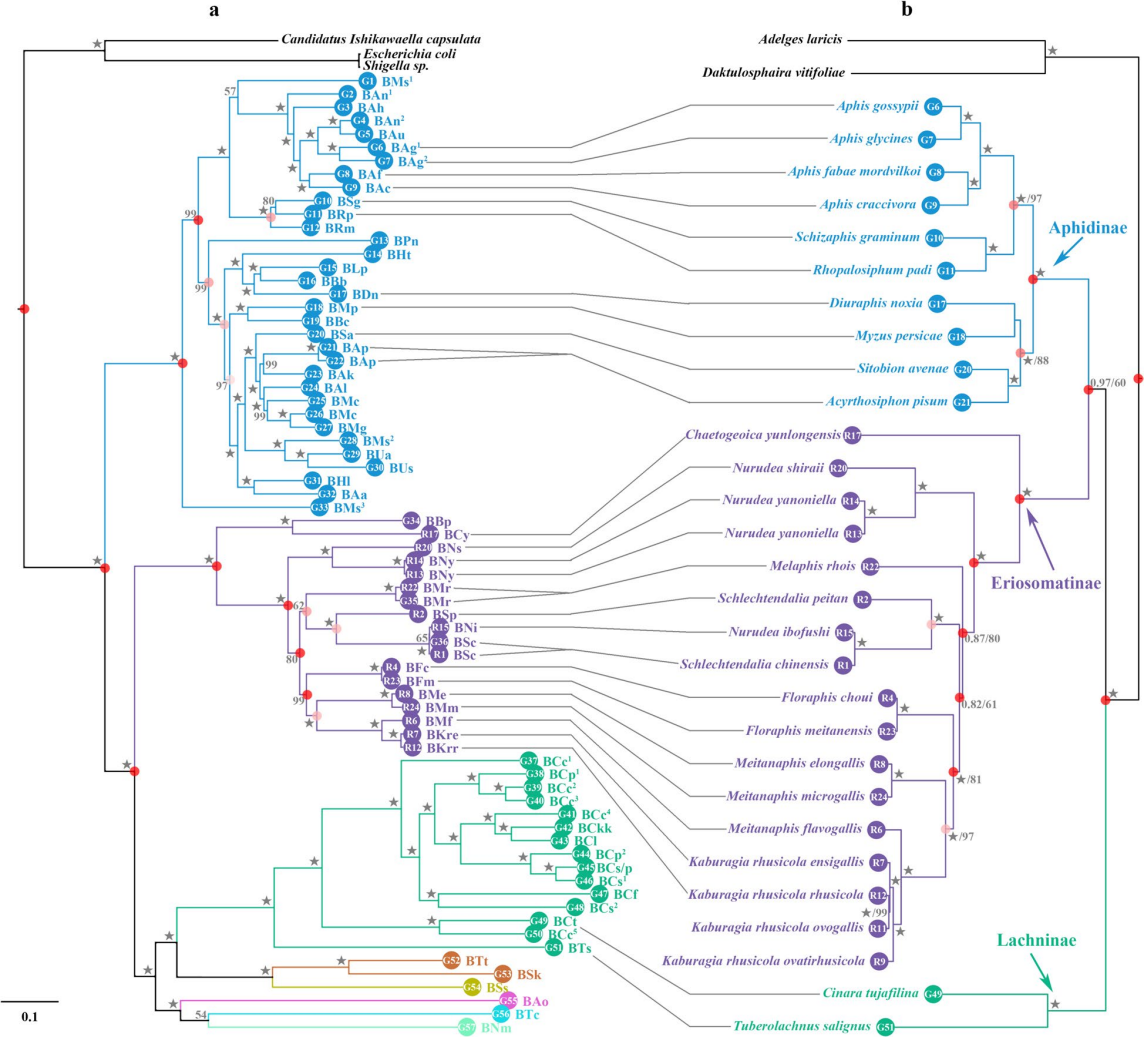
Tanglegram depicting the associations of *Buchnera* (**a**) and host aphids (**b**) by TreeMap analysis. Stars at the branches represent bootstrap support of ML 100% and BI 1.00. The red dot on the internal node represents the significance of their congruence according to the TreeMap analysis with darker red being of greater congruence. The colors correspond to the subfamilies of host aphids represented and their respective parasite species, Blue: Aphidinae, Purple: Eriosomatinae, Green: Lachninae, Brown: Calaphidinae, Yellow: Phyllaphidinae, Pink: Anoeciinae, Cyan: Thelaxinae, Light green: Hormaphidinae. Code of *Buchnera aphidicola* are the same as Table 1 and Additional file 1: Table S1

**Fig. 2:**
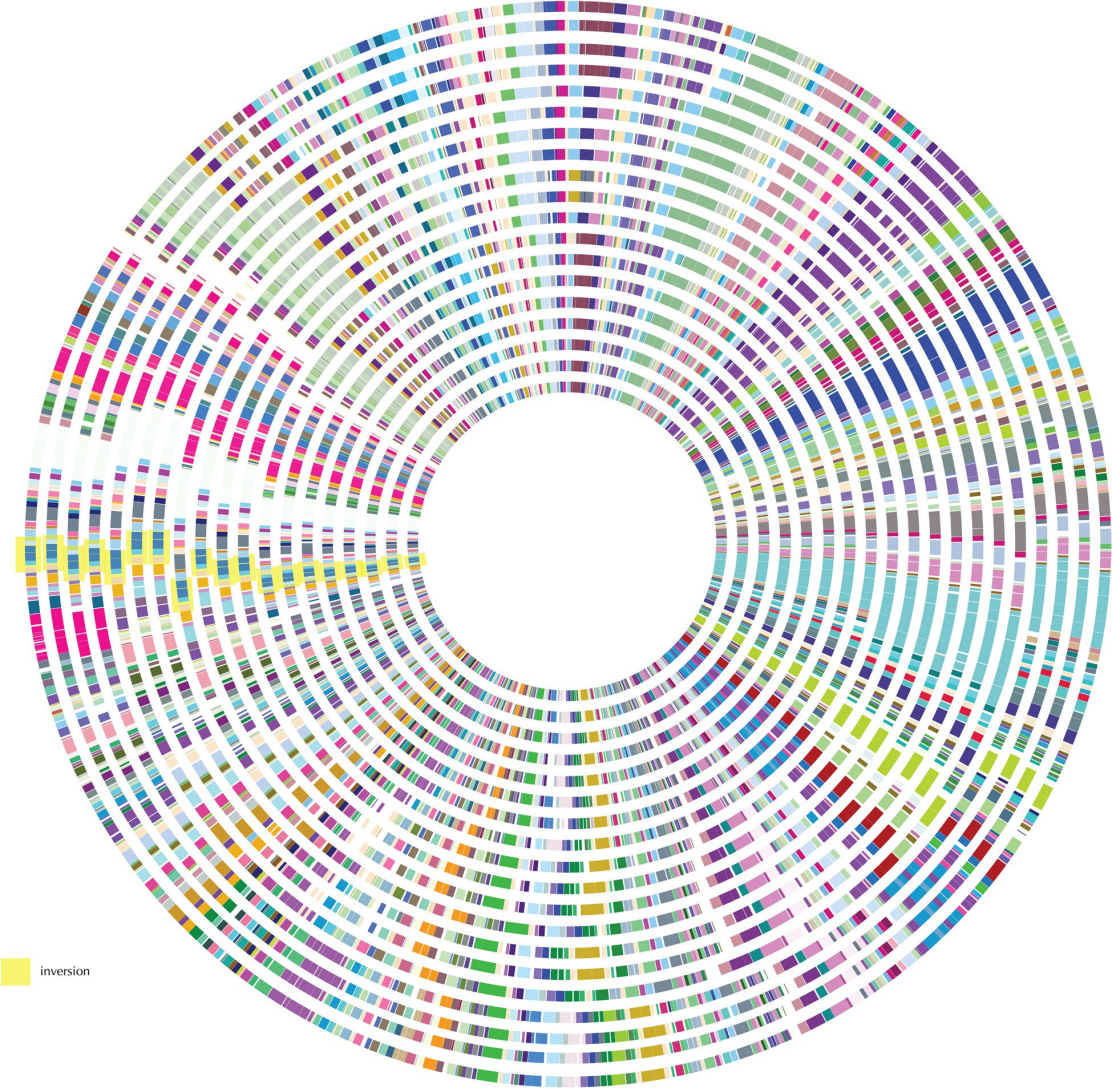
Genomes in circular genome diagram (outer to inner, organized in phylogenetic order). Aphid hosts: *S. graminum*, *M. persicae*, *U. ambrosiae*, *A. pisum*, *T. salignus*, *C. cofinis*, *C. tujafilina*, *C. cedri*, *C. pseudotaxifoliae*, *C. strobi*, *C. fornacula*, *B. pistaciae*, R20 (*N. shiraii*), R22 (*M. rhois*), R1 (*S. chinensis*), *S. chinensis*, R4 (*F. choui*), R8 (*M. elongallis*), and R7 (*K. r. ensigallis*)

Based the concatenated protein-coding gene dataset, we constructed a maximum likelihood (ML) phylogenetic tree for all the 72 *Buchnera* samples with *Escherichia coli*, *Shigella* sp., and *Candidatus Ishikawaella capsulata* as outgroups (Fig. 1a). The tree well supported the monophyly of each subfamily sampled in this study. We observed a seven-gene (*thyA*, *lgt*, *lysA*, *lysS*, *lysU*, *prfB*, and *ygfZ*) inversion across all *Buchnera* genomes within the subfamily “Eriosomatinae” clade (Fig. 2 and Table 2), and we also found a four-gene (*leuA*, *leuB*, *leuC*, and *leuD*) rearrangement with three different orders in different subfamilies, i.e., ABCD in *Anoecia oenotherae* from the subfamily Anoeciinae, and DCBA in *Therioaphis trifolii* + *Sarucallis kahawaluokalani* + *Stegophylla* sp. from the subfamily Calaphidinae and Phyllaphidinae, respectively, while ADCB was observed in the tribe Fordini from the subfamily Eriosomatinae. Moreover, the genes *trpG* and *trpE* were inserted in a different location of the *Buchnera* genomes within Eriosomatinae and Calaphidinae + Phyllaphidinae aphids, and the genes *ibpA* and *ibpB* also presented in different positions within *Buchnera* of some aphids in subfamily Aphidinae and Calaphidinae + Phyllaphidinae. Despite stability of gene order, there has been substantial gene loss across each subfamily. *Buchnera* corresponding to each aphid subfamily had different numbers of gene losses shared that are still retained in any sampled *Buchnera* taxon within that group to date, i.e., Eriosomatinae, Calaphidinae, and Aphidinae (Table 2). There are 15 gene losses shared among all members of the “Lachninae” clade, 18 gene losses shared among the “Phyllaphidinae” clade, two gene losses shared among the “Hormaphidinae” clade, and one gene loss shared among each of the “Thelaxinae” and “Anoeciinae” clades.

**Table 2:** Unambiguous and unreversed gene gains, losses, and rearrangements across the *Buchnera* tree. Asterisk indicate genes highlighted as transporters that show patterns of loss in *Buchnera* lineages in Charles 2011 [56]

Subfamily	Gene lost	Gene gain	Gene inversion	Gene rearrangement
Lachninae	atpA*, atpB*, atpC*, atpD*, atpE*, atpF*, atpG*, *bioB*, crr*, ptsG*, ptsH*, ptsI*, *purA*, *purB*, *purH*	-	-	-
Eriosomatinae	-	pal*	*thyA*, *lgt*, *lysA*, *lysS*, *lysU*, *prfB*, *ygfZ*	*leuA*, *leuD*, *leuC*, *leuB*, *trpE*, *trpG*
Calaphidinae	-	-	-	*leuD*, *leuC*, *leuB*, *leuA*, *trpE*, *trpG*, *ibpA*, *ibpB*
Phyllaphidinae	*ftsY*, fliF*, fliP*, *fabB*, *rnt*, *fabZ*, flhB*, flhA*, *argS*, *acpS*, lepB*, *fabI*, *rnb*, *tpiA*, *fabD*, *fabG*, *polA*, *lon*	*ydiN*	-	*leuD*, *leuC*, *leuB*,* leuA*, *trpE*, *trpG*, *ibpA*
Hormaphidinae	*erpA*, *truB*	*lapA*	-	-
Thelaxinae	*mscS*	-	-	-
Aphidinae	-	secB* flgK*	-	*ibpA*, *ibpB*
Anoeciinae	*cyoA*, *nfuA*	-	-	*leuA*, *leuB*, *leuC*, *leuD*
Calaphidinae + Phyllaphidinae	*cca*, *nfo*, *pth*, *suhB*, *recC*, *recB*, *recD*	-	-	-
Calaphidinae + Phyllaphidinae + Lachninae	*gmk*	-	-	-
Hormaphidinae + Thelaxinae	-	-	-	-
Hormaphidinae + Thelaxinae + Anoeciinae	-	-	-	-
Hormaphidinae + Thelaxinae + Anoeciinae + Calaphidinae + Phyllaphidinae + Lachninae	*hflC*, *hflK*, pitA*	-	-	-

**Table 3:** Information of the family Aphididae species and outgroup downloaded from GenBank

Subfamily	Tribe	Species	Accession no			
			mt	EF	LWO	18S
Aphidinae	Aphidini	*Aphis craccivora*	KX447142	DQ493842	-	-
		*Aphis fabae mordvilkoi*	MG897128	AY219724		
		*Aphis gossypii*	KJ669654	EF640162	-	KF018922
		*Aphis glycines*	KC840675	EU358911	KM501301	EU035497
		*Rhopalosiphum padi*	KT447631	AY219719	FM177114	AF487718
		*Schizaphis graminum*	AY531391	JF968544	-	AH003128
	Macrosiphini	*Diuraphis noxia*	KF636758	DQ005144	-	-
		*Myzus persicae*	KU236024	EF419315	AJ489282	AF487712
		*Sitobion avenae*	KJ742384	DQ005155		
		*Acyrthosiphon pisum*	FJ411411	AF068480	AJ489281	X62623
Lachninae	Tuberolachnini	*Tuberolachnus salignus*	KP722566	FM174685	FM177113	-
	Eulachnini	*Cinara tujafilina*	KP722583	FM174684	KM501231	-
Outgroup		*Daktulosphaira vitifoliae*	DQ021446	FM174707	AJ489295	-
		*Adelges laricis*	KP722589	DQ493827	-	-

**Fig. 3:**
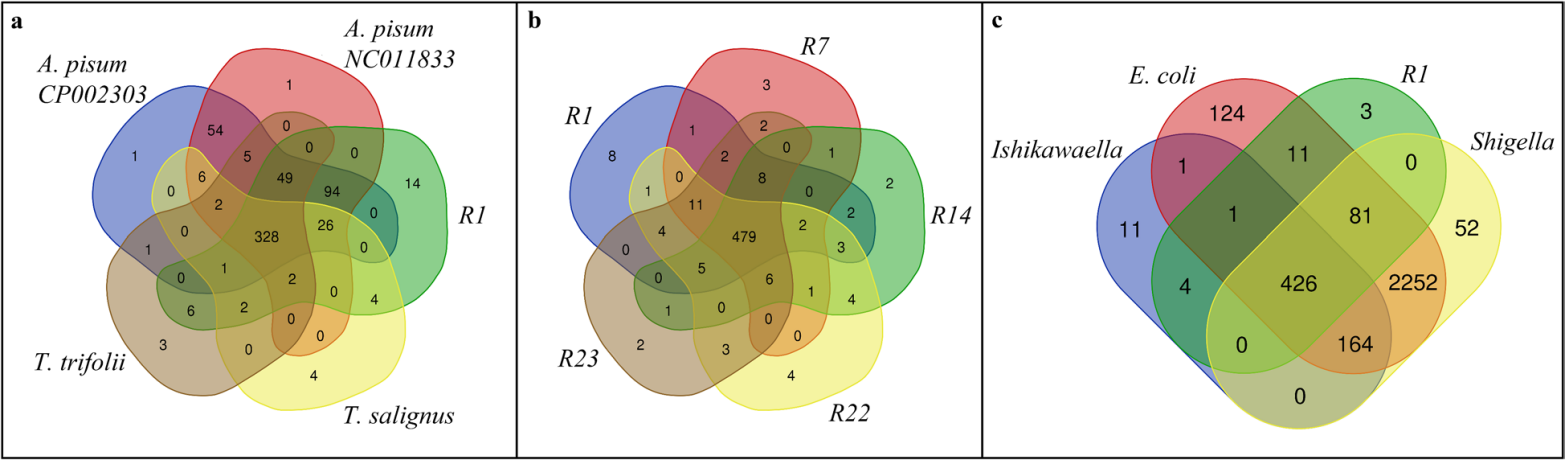
Venn diagrams showing shared gene content of *Buchnera* samples. **a** Five representatives from the ingroup. **b** Five representatives from *Rhus* gall aphid species (purple clade in Fig. 1). **c** Three outgroup species and one ingroup taxon

Genomes of the outgroup taxa, *Escherichia coli* (4.6 Mbp) and *Shigella* sp. PAMC 28760 (4.5 Mbp), are almost ten times as large as any of the genomes of *Buchnera*. *Escherichia coli* and *Shigella* sp. contain all the genes that are part of the *Buchnera* pan-genome, which has 684 genes. *Ishikawaella* also has a reduced genome (700 Kbp) but does not have the conserved gene order that the *Buchnera* genomes have nor does the *Ishikawaella* genome contain all genes of the *Buchnera* pan genome (Fig. 3), indicating that it has its own history of gene loss. We identified a core genome of 256 shared genes in the sampled species of *Buchnera*. However, the high level of nucleotide sequence divergence shows that some putatively homologous genes do not align at the nucleotide level. The *atp* gene family, which contains up to eight members (*atpA-atpH*), is notably absent from all members of the “Lachninae” clade, as is the *mur* gene family (*murA*-*murD*, *murF*, *murG*, and *murI*). GC content (Table 1 and Additional file 1: Table S1) for *Buchnera* samples ranged from 20.1 to 28.1%. For *E. coli* and *Shigella*, GC content are both 50.8%, but 30.2% for *Ishikawaella*.

The RAxML tree showed three major clades of *Buchnera* with high support corresponding to the subfamilies of aphid hosts (Fig. 1a). The relationship among the *Buchnera* strains in different aphid subfamilies is as follows: *Buchnera* in—(Aphidinae, (Eriosomatinae, (Lachninae + Anoeciinae + Thelaxinae + Hormaphidinae + Phyllaphidinae + Calaphidinae))), to which we referred as the *Buchnera*-Aphidinae, Eriosomatinae, and LATHPC clades, respectively. In the case of the LATHPC clade, our sampling comprised only one or two genomes for each of the five subfamilies except for Lachninae, and there were some bacterial taxa for which there were no corresponding aphid sequences available. The branching patterns of the *Buchnera* tree completely correspond to the classification of the aphids (Fig. 1a).

### Aphid phylogenetics

Our samples consisted of three subfamilies and 31 species from the family Aphididae with two species from the two families Phylloxeridae and Adelgidae as outgroups (Table 3). The combined, aligned mitochondrial genomes (13 protein-coding genes and two rRNAs) represented 13,159 bp and the three nuclear genes comprised a matrix of 4,531 bp in length. We concatenated the 15 mtDNA genes with the three nuclear datasets based on the results of an incongruence length difference (ILD) test (*p* = 0.33 > 0.01) and the concatenated sequences had 17,690 bp.

Independent analyses of the concatenation of the three nuclear genes and mitochondrial genes yielded topologies with high support for clades, especially comprising species within the same genus, but the relationships among deep nodes had low bootstrap support (BS < 60). The four phylogenetic analyses, i.e., ML and Bayesian inference (BI) with and without partitioning, of the concatenated, or total-evidence, dataset showed high support for the monophyly of all three subfamilies (Aphidinae, Eriosomatinae, and Lachninae) by the Bayesian posterior probabilities (PP) and BS (BI-PP = 1.00 and ML-BS = 100), and the tribes within each subfamily, except for Tuberolachnini and Enlachnini in Lachninae with only one species used in our sampling, i.e., *Tuberolachnus salignus* and *Cinara tujafilina*. In all trees, the six genera of Melaphidina aphids composed five generally well-supported clades: *Nurudea*, *Melaphis*, *Schlechtendalia*, *Floraphis*, and *Meitanaphis* + *Kaburagia*. There was some incongruence among the concatenated trees generated using the four different approaches. Notably, Lachninae was sister to a clade of Aphidinae and Eriosomatinae in all trees with high support (BI-PP = 1.00 and ML-BS = 100) except the ML tree with no partitioning, in which Lachninae and Aphidinae were sisters but with low support (ML-BS = 59). Our results also yielded differences in relationships within the highly supported clade of *Acyrthosiphon* + *Sitobion*, *Diuraphis*, and *Myzus* (BI-PP = 1.0 and ML-BS > 88) (Additional file 2: Figs. S1, S2 and S3).

Therefore, we showed the BI tree resulting from the analysis with partitioning among genes (Fig. 1b). Notably, the Bayesian trees reconstructed under the GTR + G model may be more robust estimates of relationships and evolutionary distances due to base compositional biases inferred by IQTree [57]. The BI topology showed that the three subfamilies formed three clades with well-support (BI-PP = 1.00 and ML-BS = 100), and Aphidinae is closer to Eriosomatinae than to Lachninae with high BI-PP = 0.97, but low ML-BS = 60 (Fig. 1b). The four species *Acyrthosiphon pisum*, *Sitobion avenae*, *Myzus persicae*, and *Diuraphis noxia* of the tribe Macrosiphini grouped as a clade with high support as well as the six species of the tribe Aphidini, whose monophyly was well supported (BI-PP = 1.00 and ML-BS = 97). The two tribes Tuberolachnini and Enlachnini only include one species *Tuberolachnus salignus* and *Cinara tujafilina*, respectively, which grouped as a high-supported clade.

### Cophylogenetic analyses of Aphid-*Buchnera*

**Fig. 4:**
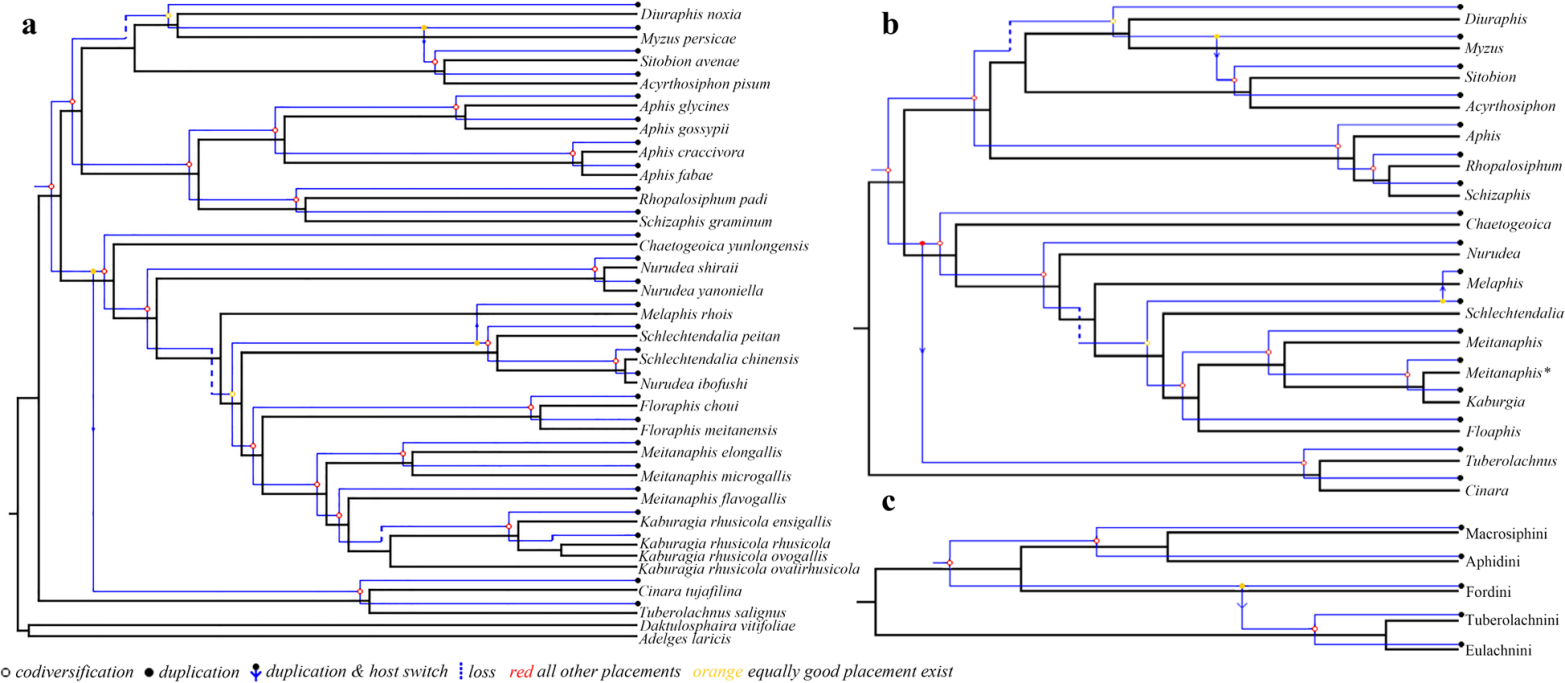
The cophylogeny of aphid and *Buchnera* from Jane at the different level with the reconciled trees based on the aphid tree (BI) and *Buchnera* tree (ML): **a** species; **b** genus; **c** tribe. Black and blue maps indicate the phylogenies of the aphid and *Buchnera*, respectively. Hollow red circles indicate co-diversification events; solid red and yellow circles indicate duplications; arrows indicate host switch events; dotted lines indicate loss events

Based on topology-based cophylogenetic analyses by TreeMap and Jane, evolution of *Buchnera* was generally in accordance with their aphid hosts at individual, species, generic, and tribal levels (Figs. 1 and 4 and Table 4). A disagreement between trees results from the position of the subfamily Lachninae (Fig. 1), which is sister to the group of Aphidinae + Eriosomatinae in the aphid tree (BI-PP = 1.00 and ML-BS = 100), but *Buchnera* in Lachninae is closer to the *Buchnera* species in Eriosomatinae in the bacterial tree. We detected three duplication and host switch events between *Myzus* and *Acyrthosiphon* + *Sitobion*, *Melaphis* and *Schlechtendalia*, two genera of Lachninae, which group was well supported (100%) in both the *Buchnera* and aphid tree. Also, one duplication and host switch event was detected between the two genera *Melaphis* and *Schlechtendalia* species at the genus level in the tribe Fordini.

**Table 4:** The parameters obtained from event-based cophylogenetic analysis with the programs TreeMap and Jane

Level	Treemap					Jane			
	C	D	L	H	S	C	D	DH	L
Individual	44	12	12	0	24	23	4	3	4
Species	44	6	11	0	17	22	0	3	6
Genus	26	6	9	0	15	13	0	3	2
Tribe	6	2	3	0	5	3	0	1	0
Fordini	24	2	5	0	7	12	0	1	3

*C* co-diversification, *D* duplication, *L* loss, *H* host switch, *S* sorting events, *DH* duplication and host switch

**Fig. 5:**
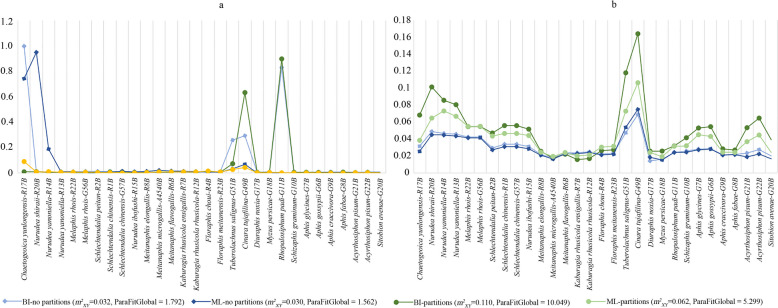
The cophylogeny signal of individual aphid-*Buchnera* associations from ParaFit (**a**) and PACo (**b**)

The distance-based methods for cophylogenetic inference showed significant global fit for all four reconstructions of the aphid tree compared with the bacterial tree (*p* = 0.001 for ParaFit and *p* ≤ 0.001 for PACo), and the signal of global congruence was significant (Fig. 5). However, the analyses based on the aphid trees without partitioning showed considerably better global fit with the tree of bacteria than the analyses based on trees reconstructed with partitioning. For both ParaFit and PACo, the ML analyses without partitions yielded the best fit with the bacterial tree, while BI with partitions showed the least good fit. In the PACo, trees reconstructed without partitions led to associations showing slightly positive effects on the global fit, while analyses with partitions showed much larger negative effects on global fit. Each individual associations were analyzed by the individual host–parasite (H-P) link test in ParaFit. Both ML and BI analyses with and without partitions showed 24 of the 30 aphid-*Buchnera* links and had a significant coevolution relationship with ParaFit1 or ParaFit2 (*p* value ≤ 0.05). The largest Aphids-*Buchnera* residuals in all the four PACo analyses were *Cinara tujafilina* and *Tuberolachnus salignus*, indicating that these two species had the weakest signal of cophylogeny with their symbiont *Buchnera*.

## Discussion

### *Buchnera* comparative genomics

*Buchnera* is endosymbiotic within host species throughout the aphid lineage. Even though *Buchnera* is recognized to include a single species, *B. aphidicola*, it exhibits extremely high genetic diversity as a lineage of Gammaproteobacteria and is at least 50 million years old based on the *Buchnera* accessions studied so far [19, 38, 58, 59]. While there has been substantial evolutionary change in *Buchnera* (see Additional file 3: Dataset S1 for pairwise identity across homologous regions) with gene losses and gains (Table 2 and Additional file 3: Dataset S1), the arrangement of genes appears extremely stable (cite our data) [59]. In prior studies, two rearrangements and one inversion were detected in the sequenced genomes of *Buchnera* in Eriosomatinae, Calaphidinae, Phyllaphidinae, and Anoeciinae [15, 35, 38, 60–63]. In this study, we found rearrangement of *ibpA* and *ibpB* in Calaphidinae + Phyllaphidinae, despite sampling one or two species from each of the subfamilies Calaphidinae, Phyllaphidinae, and Anoeciinae. 

Our analysis on the *Buchnera* pan-genome for 72 taxa contained 684 genes, and the core genome contained 256 genes. We calculated gene gains and losses of each clade by comparing with core genome of other clades, rather than with the inferred ancestral genome. We investigated gene gains by comparing the *Buchnera* core genomes comprising only those genes shared by all sampled species to pan-genomes of other clades. To reconstruct the ancestral genome of *Buchnera*, van Ham et al. (2003) identified 601 ancestral protein-coding genes based on three genomes of *Buchnera* [35], while Chong et al. (2019) inferred 616 ancestral protein-coding genes based on 39 complete genomes [38]. Although we have expanded the sampling of *Buchnera* compared to these prior studies, there are many unsampled or as yet unknown strains of *Buchnera*. van Ham et al. (2003) suggested that gene loss in *Buchnera* continued among extant lineages at a slower rate, but there is no evidence that the gene gains are occurring [35]. Here, we found up to five gene gains based on each subfamily clade, and they all exist in *Escherichia coli* genome, four of which have the same relative positions as in *Buchnera* genome, while only the position of the *ydiN* gene showed obvious rearrangement comparing with *E. coli*. Therefore, we speculated that the gained four genes in different subfamily were relative to their loss in other subfamilies. The rearrangement of *ydiN* gene might be from regaining through loss, horizontal gene transfer from the host or other bacteria, etc. However, the evidence suggests that genes related to leucine (*leuABCD*) and tryptophan (*trpEG*) biosynthesis have undergone multiple movements between plasmid and chromosome locations, finding on the variable locations of these genes in previous and this study [15, 38, 60–62].

We performed a comparative genomic analysis of *Escherichia coli* and *Shigella* sp. PAMC 28760 representing related free-living bacteria, which were also belong to the family Enterobacteriaceae. The genome length of *Buchnera* in Aphididae hosts is much smaller than that of the two genomes of* E. coli* (4.6 Mbp) and *Shigella* (4.5 Mbp), which points to the very distinct evolutionary trajectory of these bacteria towards genome reduction [15, 35, 38]. It has been reported that *Buchnera* has some of the smallest genomes in bacteria sequenced to date and is lacking many of the genes that would enable them to live freely and, thus, be cultured. For example, Charles et al. (2011) investigated the reduction of membrane transporter genes in *Buchnera*, which are essential for shared metabolic networks [56]. These authors showed that, overall, *Buchnera* had lost membrane transporter genes, but the decreased diversity and limited substrate specificity of those retained probably facilitated essential movement of metabolic products. Moreover, genes involved in heat tolerance may have been purged from the genomes of *Buchnera*, yielding a greater heat sensitivity, which may, in turn, constrain the ecological and geographic ranges of the aphid hosts [64].

In this study, we included the genomes of the same four strains studied by Charles et al. (2011; *A. pisum*, *S. graminum*, *B. pistaciae*, and *C. cedri*) [56]. What is striking is that the genes lost in *Buchnera* of the aphid subfamily Lachninae (or the “Lachninae” clade) are present in all the other *Buchnera* lineages sampled in this study. Within the “Lachninae” *Buchnera*, the most of genes lost have protein products that are membrane transporters. These include (1) the F-ATP synthase complex, revealing that these *Buchnera* do not use this pathway to drive ATP synthesis; (2) members of the ATP-dependent *mur* ligases (*murA-murD*, *murF*, *murG*, *murI*), which are key in the synthesis of peptidoglycan, or the bacterial cell walls [65]; (3) the glucose/mannitol phosphotransfer-driven group translocators superfamily (PTS) transport system, which could necessitate that *Buchnera* import carbon sources to build up essential amino-acid backbones for its host from other transporters. In contrast, *Buchnera* strains from other aphid subfamilies possess all complete PTS systems for the importation of glucose and mannitol, which are the two main carbon sources detected in the cytoplasm of bacteriocytes [66].

We especially found the peptidoglycan-associated lipoprotein (*Pal*) encoded only in the genome of *Buchnera* from the aphid subfamily Eriosomatinae in our current study. The protein *Pal* was anchored in the outer membrane (OM) of Gram-negative bacteria and interacts with *Tol* proteins [56]. But no inner membrane *Tol* proteins have been found in any strains of *Buchnera*. The role of *Pal* protein in Eriosomatinae remains unknown, but its features suggested the possibility of neo-functionalization.

More work is needed to include other lineages related to *Buchnera* and *Candidatus Ishikawaella capsulata*, a special intestinal bacterium and a sister group of the obligate intracellular symbiote *Buchnera* in aphids [38], that might be able to tell us more about the evolutionary processes leading to their acquisition as a symbiont and genome reduction. Gene repertoire and elevated evolutionary rate of *Ishikawaella* were strikingly similar to *Buchnera* [67], providing a possible case of similar evolutionary patterns and convergence of some gene. This work represents one of several recent and ongoing efforts to expand the number of sequenced genomes of *Buchnera* (i.e., 72 included in this study).

### Call for *Buchnera* taxonomic delimitations

The large number of available genomes represents considerable genetic resources for taxonomic revision of *Buchnera*. In cases where bacteria cannot be cultured, a description may serve as the type according to Rule 18a of the International Code of Nomenclature of Bacteria [68], and, notably, this rule was cited in the original description of *Buchnera aphidicola* [20]. The genomic data for *Buchnera* could be utilized as the basis for delimiting species, even though the breadth of genomic variation allowable within a single bacterial species may be much larger than that in eukaryotes [69], depending on the species concept [70].

Now that there are a sizable number of sequenced genomes of *Buchnera* (i.e., 72 included in this study), we call for a reassessment and further studies on the taxonomy of the group. Such taxonomic reassessment and debate may result in an expansion of the number of species within *Buchnera*. The reassessment needs to further expand the taxon sampling and it is possible that other taxonomically divergent relatives of *Buchnera* may be discovered (with genomes reduced intermediately between *E. coli* and *Buchnera*).

### Aphid and *Buchnera* co-evolution

As an obligate endosymbiont, *Buchnera aphidicola* provides aphid hosts with several essential nutrients, and numerous studies have indicated the congruent phylogenetic relationship between the endosymbiont and the aphids [19, 22, 71, 72]. Most of previous studies focused on lower taxonomic levels and/or several genes (e.g., closely related species or intraspecific lineages). Here, we constructed the phylogenetic relationship with the broadest taxonomic sampling of whole-genome sequences across *Buchnera* on diverse host lineages and of complete mitochondrial genome and three nuclear genes across the host aphids (Hemiptera: Aphididae).

The cophylogenetic analyses demonstrated significant patterns of coevolution between *Buchnera* and its aphid hosts. However, the patterns are more complex than simply shared branching order. In fact, the most robustly reconstructed evolutionary trees for aphids using data partitioning [73] compared to the bacterial tree showed significant coevolutionary relationships (Table 4 and Figs. 1, 4, and 5), but analyses for individual associations showed negative (and often significant) impacts on global fit, which have a higher value than most individual associations of no-partitioned data. Prior studies have regarded this complex specific relationship inferred using distance-based cophylogenetic methods as representing one or more host switches [74, 75].

However, this seems unlikely in our case as visual inspection of the aphid and bacterial trees with TreeMap (Fig. 1b) suggests that branching orders among the hosts and endosymbionts are highly congruent. Thus, the significantly negative relationships may reflect differing, non-random tempos of evolution that are detectable using the distance-based methods. In particular, a large number of gene losses in *Buchnera* in comparison with phylogeny of interspecific aphids has also suggested that bacterial evolution may be fast while that in aphid may be slow and vice versa [45]. While testing this hypothesis will necessitate dated phylogenies, it is logical in that shared periods of rapid evolution could make the shared metabolic network unstable and, thus, be maladaptive [76]. Somewhat similarly, temporal patterns may emerge as rapid evolution in one symbiotic partner triggers rapid evolution in the other, constantly perpetuating and stabilizing the fragile obligate relationship on a dynamic evolutionary landscape [77].

The topology of *Buchnera* was somewhat inconsistent with that of aphid hosts, which implied that duplication and host switch events might happen. Actually, we examined and found that *Melaphis* and *Schlechtendalia* with their *Buchnera* had duplication and host switch events in the group of *Rhus* gall aphids. Additionally, the strong support in the subfamily Aphidinae detected one duplication and host switch events between *Myzus* and *Acyrthosiphon* + *Sitobion*. These aphids exhibited both sexual and parthenogenetic reproduction [49]. It is speculated that the “duplication and host switch” of *Buchnera* may occur through hybridization during sexual reproduction in aphids. Overall, our results highlight the complexity of the obligate symbiotic relationship between aphids and bacteria and have generated coevolutionary hypothesis for this intriguing system that merit additional studies.

## Methods

*Buchnera aphidicola* as the endosymbiotic bacteria has never been successfully cultured. Thus, we employed high-throughput genome skimming sequencing technology to simultaneously generate sequence data for the host aphids and endosymbiotic *Buchnera*.

### Isolation and sequencing of genome DNA

We collected galls on *Rhus* from China except for the galls of the North American aphid species, *Melaphis rhois*, which we collected from the USA (Table 1). Aphids from the same gall represent parthenogenetic clones, and these comprise a sample. We stored samples from each gall in 75% ethanol and 100% ethanol for identification and DNA extraction purposes, respectively. Our 17 samples of the *Rhus* gall aphids included all six genera and the 13 species recognized within Melaphidina [5, 47, 48]*.* We also collected and sampled galls of *Chaetogeoica yunlongensis* representing the sister subtribe Fordina collected from the host plant *Pistacia chinensis*. We deposited voucher specimens at the School of Life Science of Shanxi University in China.

We extracted genomic DNA from five aphid individuals within each sampled *Rhus* gall by immersing them in distilled water for at least 36 h and then using a DNeasy extraction kit (QIAGEN, Valencia, CA). Our extractions for the aphids also included the DNA of the endosymbiotic bacteria. We quantified the total DNA yields and assessed quality using NanoDrop-2000 (Thermo Fisher Scientific) before sending the samples partially to the Genomic Sequencing and Analysis Facility (GSAF), University of Texas, Austin, for library construction and genomic sequencing. DNAs were sheared into ~ 500 bp fragments using the Covaris M220 Focused-ultrasonicator to prepare the library with a TruSeq Nano DNA library preparation kit (Illumina, FC-121–4003). Genomic sequencing comprised paired-end reads of 2 × 150 bp generated on an Illumina NextSeq sequencer with an insert size of 400 bp. We filtered the raw data using Trimmomatic v. 0.35 with default settings (ILLUMINACLIP:Tru Seq3-PE:2:30:10 LEADING:3 TRAILING:3 SLIDINGWINDOW:4:15 MINLEN:36) [78].

### *Buchnera* genomics and phylogenomics

We assembled shotgun sequence data using SPAdes v. 3.7.1 [79] with kmers 21, 33, 55, 77, 99, and 127. Since we used the whole body of aphid individuals to extract the genomic DNA and sequenced, also with the abundance of symbionts *Buchnera* in aphid cells, these assemblies included the complete genomes of the bacterial endosymbionts. For those genomes whose contiguous circular genomes were not able to be completely generated but with several contigs, we used the mapping function in Geneious to filter out the aphid contigs by referring to the closest complete *Buchnera* genomes [80]. For gene counting comparisons, contigs were arranged in the most possible conservative way, i.e., orientation was assumed to be collinear with reference to the complete single circular contig genomes. We annotated the bacterial genomes with Prokka v. 1.12 [81].

For comparative genomic and co-phylogenetic analysis, we downloaded complete genomes of *Buchnera* from GenBank with corresponding aphid sequences. In total, there are 73 genomes of *Buchnera aphidicola* in 56 species of aphids reported in GenBank. Usually, there were the similar genomic organization for the same species, so we compared and selected one genome of *Buchnera aphidicola* in each aphid species except for the aphid *Acyrthosiphon pisum*, which published two genomes but with relative high variability (GenBank accession no. CP002303 and NC_011833). All the 57 publicly available genome sequences and our dataset of 15 newly sequenced genomes were shown in Additional file 1: Table S1. The gene losses and gains were counted with respect to the *Buchnera* core genome and pan-genome. We chose this method because there are almost certainly unsampled or yet unknown taxa, which would be important to consider if we were to calculate the gene history of *Buchnera* with reference to its sister taxon. We also performed a comparative genomic analysis of *Buchnera* with three outgroups: *Escherichia coli*, *Shigella* sp. PAMC 28760 (representing related free-living bacteria), and *Candidatus Ishikawaella capsulata* (a special intestinal bacterium and a sister group of the obligate intracellular symbiote *Buchnera* of aphids [38]).

We performed phylogenetic analyses using 144 protein-coding genes that were present in the genomes of all the 75 *Buchnera* species and three outgroup taxa [40]. Each protein-coding gene dataset was aligned using MAFFT version 7 [82] with Translation Align implemented in Geneious 10.2.4 with default settings. We generated partitioning schemes for these shared genes in PartitionFinder2 [83, 84] and performed a partitioned analysis in RAxML v. 8.2.7 [85] under the GTRGAMMA model with 1000 full bootstrap replicates. We also used the bacterial sequence data to generate Venn diagrams of genome content with a web-tool from the University of Gent (http://bioinformatics.psb.ugent.be/webtools/Venn/), and we visualized circular genome plots using CGView [86].

### Aphid genes and phylogenetic analysis

We constructed the aphid phylogeny by using the 17 mitogenomes of Fordini species sampled in this study (16 *Rhus* gall aphids and one species *Chaetogeoica yunlongensis* living on *Pistacia chinensis*) and combining the mitogenomes from GenBank representing 12 additional species of the family Aphididae, belonging to two subfamilies, Aphidinae and Lachninae, and four tribes: Aphidini, Macrosiphini, Tuberolachnini and Eulachnini. We used two species, *Daktulosphaira vitifoliae* and *Adelges laricis*, from the other aphid superfamilies, Phylloxeroidea and Adelgoidea, as outgroups. We also obtained the three nuclear markers, *18S*, *EF-1α*, and *LWO* from the clean reads of the shotgun skimming genome by remapping the reads to alignments of available sequences in GenBank and also performing de novo re-assemblies of reads in SPAdes for checking and correcting the degenerate bases in our previous report [53]. Thus, the reconstructed phylogenetic relationships of host aphids included 31 samples from 30 species, including six genera and 13 species, and employed 15 mitochondrial and three nuclear genes (Table 3).

We extracted 13 protein-coding genes (*COI*-*COIII*, *ATP6*, *ATP8*, *ND1-ND6*, *ND4L*, *Cytb*) and two rRNA loci (*12S* and *16S*) from all the aphid mitogenomes and performed the alignments by the program MAFFT v7.2 as well as the nuclear sequences [82].

The mitochondrial genes are maternally inherited and represent a non-recombining locus, so we concatenated them as a dataset. We used the partition homogeneity test [87] in PAUP* [88] to determine the appropriateness of combining the mitochondrial and nuclear genes as one dataset. Based on the above test (ILD, *p* = 0.33 > 0.01), we concatenated the 15 mitochondrial genes and the three nuclear genes into a total-evidence matrix. We analyzed the total-evidence matrix in four ways by using the ML and BI with and without partitioning by gene. We performed the ML analyses in IQTree [89] with internal model selection based on ModelFinder [90] according to the Bayesian Information Criterion. We accomplished BIs for all individual genes, the combined nuclear genes, and the combined mitochondrial genes using MrBayes v. 3.2.3 [91, 92] on the high-performance computing cluster available via the Cipres Science Gateway (https://www.phylo.org). The BIs with and without partitioning by gene each consisted of two simultaneous, independent runs of 10 million generations of one cold chain and five hot chains with sampling every 1000 generations. For BI, we applied the GTR + G model [93], which may be more robust to base compositional biases inferred in IQTree [57]. Following BI, we examined the output in Tracer v. 1.6 [94] to verify that simultaneous runs had each reached stationarity according to effective sample sizes (ESS) > 200 and to assure the suitability of a 10% burnin. We performed the 10% burnins and combined trees from simultaneous BI runs in Log Combiner, and we summarized the trees on the maximum clade credibility tree with median branch lengths using Tree Annotator, both of the BEAST v. 1.8 package [95].

### Cophylogenetic analyses of the endosymbiont *Buchnera* and its host aphids

To determine the degree to what aphids and their endosymbiotic bacteria have coevolved, we performed cophylogenetic analyses based on our reconstructed phylogenetic trees. Cophylogenetic analyses are either event-based and utilize tree topology or distance-based and utilize tree shape [96]. In general, distance-based methods compare distance matrices derived from tree branches transformed using principal coordinates. We performed event-based cophylogenetic analysis using TreeMap v3.0 [97] and Jane v4 [98] to determine whether there is a significant match between *Buchnera* and aphid trees at different taxonomic levels, e.g., individuals, species, genus and tribe, and what is the best explanation for any differences between the two trees.

In TreeMap, the algorithm maximizes the number of both co-diversification events and evolutionary events regarded as more complex, such as diversification of the parasite without diversification in the host (duplication), diversification of the host without diversification in the parasite (“failure to diverge”), loss of the host-parasite association (loss), and host switching. Within TreeMap, we tested the least costly reconciliation against a number of randomly generated trees to determine if the result obtained is statistically significant.

We ran Jane v4 to determine the coevolutionary events among co-diversification and duplication, loss, failure to diverge, and duplication followed by host switching likely accounted for the patterns of *Buchnera*-aphid associations. This analysis assigns a range of costs to each coevolutionary event and attempts to identify the most parsimonious solution that minimizes the costs. The event-costs that we used in running Jane were co-speciation = 0, duplication = 1, duplication and host switching = 2, loss = 1, failure to diverge = 1.

We performed distance-based cophylogenetic analyses in ParaFit [26] implemented in the ape v.5.3 package [99] for R and PACo v.0.3.2 [100] implemented as a R package [101] using the BI tree resulting from the analysis with partitioning among genes. ParaFit and PACo differed in how they made comparisons between the distance matrices of the two organismal partners [100]. For this analysis, our input data included only bacterial species in aphid hosts represented in the aphid phylogeny, and all other aphids were removed from the distance matrices. In ParaFit, we applied a “lingoes” correction [102] to negative eigenvalues within distance matrices transformed as principal coordinates, and we used the transformed and corrected matrices to determine global fit between the aphid and bacterial trees. We tested the significance of the fit using 999 permutations comprising randomization of the associations between aphid and bacterial species. ParaFit assesses the significance of each H-P association, using the ParaFitLink1 and ParaFitLink2 statistic. For PACo, we performed a square root transformation of the bacterial distance matrix prior to obtaining principal coordinates to circumvent negative eigenvalues. We then determined global fit and measured significance using 1000 permutations of aphid-bacterial associations. PACo generates residuals of the Procrustean fit, which describes the contribution of each individual H-P association to the global fit (smaller residuals means a more congruence). We performed the ParaFit and PACo analyses on all four trees resulting from the BI and ML analyses of the combined sequence data to account for weakly supported topological incongruence outside of Melaphidina and differences in branch lengths.

### Supplementary Information

Additional file 1: Table S1 Information of *Buchnera* genomes downloaded from GenBank and re-annotated with Prokka.

Additional file 2: Figures S1-S3. Fig. S1. The BI tree of aphids without partitioning. Stars at the branches represent bootstrap support of BI 1.00. The colors correspond to the subfamilies of host aphids represented, Blue: Aphidinae, Purple: Eriosomatinae, Green: Lachninae. Fig. S2. The ML tree of aphids with partitioning. Stars at the branches represent bootstrap support of ML 100%. The colors correspond to the subfamilies of host aphids represented, Blue: Aphidinae, Purple: Eriosomatinae, Green: Lachninae. Fig. S3. The ML tree of aphids without partitioning. Stars at the branches represent bootstrap support of ML 100%. The colors correspond to the subfamilies of host aphids represented, Blue: Aphidinae, Purple: Eriosomatinae, Green: Lachninae.

Additional file 3: Dataset S1 The genes in the genome of the aphid symbiont *Buchnera aphidicola* used in this study.

## Data Availability

The raw DNA sequencing data, individual annotated genes, and complete genomes have been submitted to National Center for Biotechnology Information (NCBI) under BioProject accession PRJNA1025172 (https://www.ncbi.nlm.nih.gov/bioproject/PRJNA1025172) [103]. Full details are available in Table 1. Other *Buchnera* genomes analyzed were acquired from previous studies [14, 35–40, 52, 64, 67, 104–110] or downloaded from NCBI (https://www.ncbi.nlm.nih.gov; Additional file 1: Table S1).
