# Supplementary material for: Comparative genomics of the primary endosymbiont Buchnera aphidicola in aphid hosts and their coevolutionary relationships
Source: BMC Biol. 2024 Jun 20;22:137. doi: 10.1186/s12915-024-01934-w (PMC11188193; doi:10.1186/s12915-024-01934-w)
Supplement: Supplementary file 1 — Additional file 1: Table S1 Information of Buchnera genomes downloaded from GenBank and re-annotated with Prokka. [file 12915_2024_1934_MOESM1_ESM.docx]

Table S1 Information of *Buchnera* genomes downloaded from GenBank and re-annotated with Prokka.

| Code | Accession # | Aphid Host | Code | Genome size | # CDs | GC% | BioProject # | BioSamplet # |
| --- | --- | --- | --- | --- | --- | --- | --- | --- |
| G1 | NZ_CP029161.1 | *Melanaphis sacchari* | BMs1 | 626,137 | 581 | 25.30 | PRJNA224116 | SAMN09000090 |
| G2 | NZ_CP034885.1 | *Aphis nerii* [38] | BAn1 | 631,491 | 581 | 24.20 | PRJNA224116 | SAMN09980505 |
| G3 | NZ_CP034894.1 | *Aphis helianthi* [38] | BAh | 634,211 | 591 | 24.10 | PRJNA224116 | SAMN09980503 |
| G4 | NZ_CP034888.1 | *Aphis nasturtii* [38] | BAn2 | 630,331 | 584 | 24.80 | PRJNA224116 | SAMN09980504 |
| G5 | CP048744.1 | *Aphis urticata* [40] | BAu | 630,969 | 591 | 25.40 | PRJNA605335 | SAMN14055901 |
| G6 | NZ_CP056771.1 | *Aphis gossypii* | BAg1 | 628,098 | 580 | 25.40 | PRJNA224116 | SAMN15310392 |
| G7 | NZ_CP009253.1 | *Aphis glycines* [14] | BAg2 | 628,164 | 581 | 25.60 | PRJNA224116 | SAMN02937542 |
| G8 | NZ_CP042427.1 | *Aphis fabae* [64] | BAf | 634,931 | 589 | 24.20 | PRJNA224116 | SAMN12392394 |
| G9 | NZ_CP034897.1 | *Aphis craccivora* [38] | BAc | 632,742 | 579 | 24.40 | PRJNA224116 | SAMN09980501 |
| G10 | NZ_CP029205.1 | *Schizaphis graminum* [104] | BSg | 641,454 | 600 | 25.30 | PRJNA224116 | SAMN09004684 |
| G11 | NZ_CP034858.1 | *Rhopalosiphum padi* [38] | BRp | 643,941 | 595 | 25.20 | PRJNA224116 | SAMN09980517 |
| G12 | NZ_CP032759.1 | *Rhopalosiphum maidis* [38] | BRm | 642,929 | 602 | 25.30 | PRJNA224116 | SAMN10176941 |
| G13 | CP061275.1 | *Pentalonia nigronervosa* [105] | BPn | 617,483 | 523 | 26.10 | PRJNA628023 | SAMN16063094 |
| G14 | NZ_CP034873.1 | *Hyadaphis tataricae* [38] | BHt | 633,867 | 580 | 27.00 | PRJNA224116 | SAMN09980510 |
| G15 | NZ_CP034870.1 | *Lipaphis pseudobrassicae* [38] | BLp | 641,221 | 578 | 25.00 | PRJNA224116 | SAMN09980511 |
| G16 | NZ_CP034882.1 | *Brevicoryne brassicae* [38] | BBb | 645,850 | 596 | 25.00 | PRJNA224116 | SAMN09980507 |
| G17 | NZ_CP013259.1 | *Diuraphis noxia* | BDn | 636,266 | 586 | 25.50 | PRJNA224116 | SAMN04288104 |
| G18 | NZ_CP002701.1 | *Myzus persicae* [106] | BMp | 643,517 | 587 | 25.40 | PRJNA224116 | SAMN02641631 |
| G19 | NZ_CP034879.1 | *Brachycaudus cardui* [38] | BBc | 643,931 | 591 | 25.30 | PRJNA224116 | SAMN09980508 |
| G20 | NZ_CP034855.1 | *Sitobion avenae* [38] | BSa | 636,177 | 571 | 26.00 | PRJNA224116 | SAMN09980518 |
| G21 | NC_011833.1 | *Acyrthosiphon pisum* [107] | BAp | 642,122 | 579 | 26.30 | PRJNA224116 | SAMN02604134 |
| G22 | CP002303.1 | *Acyrthosiphon pisum* [108] | BAp | 641,771 | 734 | 26.30 | PRJNA43511 | SAMN02604268 |
| G23 | NC_017256.1 | *Acyrthosiphon kondoi* [109] | BAk | 641,794 | 581 | 25.70 | PRJNA224116 | SAMN02604329 |
| G24 | NZ_CP034891.1 | *Acyrthosiphon lactucae* [38] | BAl | 624,335 | 573 | 24.50 | PRJNA224116 | SAMN09980502 |
| G25 | CP048747.1 | *Microlophium carnosum* [40] | BMc | 642,296 | 569 | 25.60 | PRJNA605335 | SAMN14055899 |
| G26 | CP033006.1 | *Macrosiphum euphorbiae* [38] | BMe | 645,334 | 577 | 25.60 | PRJNA489301 | SAMN09980512 |
| G27 | CP034867.1 | *Macrosiphum gaurae* [38] | BMg | 643,561 | 581 | 25.90 | PRJNA489301 | SAMN09980513 |
| G28 | NZ_CP034864.1 | *Macrosiphoniella sanborni* [38] | BMs2 | 621,931 | 544 | 24.30 | PRJNA224116 | SAMN09980515 |
| G29 | NC_017259.1 | *Uroleucon ambrosiae* [109] | BUa | 615,380 | 541 | 24.10 | PRJNA224116 | SAMN02604330 |
| G30 | NZ_CP047588.1 | *Uroleucon sonchi* | BUs | 614,349 | 539 | 24.30 | PRJNA224116 | SAMN13829547 |
| G31 | NZ_CP034876.1 | *Hyperomyzus lactucae* [38] | BHl | 641,856 | 591 | 26.10 | PRJNA224116 | SAMN09980509 |
| G32 | NZ_CP034900.1 | *Artemisaphis artemisicola* [38] | BAa | 633,406 | 577 | 24.50 | PRJNA224116 | SAMN09980500 |
| G33 | NZ_CP034861.1 | *Muscaphis stroyani* [38] | BMs3 | 619,238 | 565 | 25.70 | PRJNA224116 | SAMN09980516 |
| G34 | NC_004545.1 | *Baizongia pistaciae* [35] | BBp | 615,980 | 521 | 25.30 | PRJNA224116 | SAMN02604289 |
| G35 | NZ_CP033004.1 | *Melaphis rhois* [38] | BMr | 616,452 | 542 | 25.70 | PRJNA224116 | SAMN09980514 |
| G36 | NZ_CP011299.1 | *Schlechtendalia chinensis* [52] | BSc | 607,835 | 547 | 25.80 | PRJNA224116 | SAMN03483427 |
| G37 | NC_008513.1 | *Cinara cedri* [36] | BCc1 | 416,380 | 369 | 20.10 | PRJNA224116 | SAMN02604290 |
| G38 | NZ_LR217739.1 | *Cinara piceae* | BCp1 | 435,017 | 372 | 21.90 | PRJNA224116 | SAMEA5379135 |
| G39 | NZ_LR217710.1 | *Cinara curvipes* | BCc2 | 433,837 | 376 | 21.20 | PRJNA224116 | SAMEA5379128 |
| G40 | NZ_LR217700.1 | *Cinara curtihirsuta* | BCc3 | 433,229 | 375 | 21.20 | PRJNA224116 | SAMEA5379125 |
| G41 | NZ_LR217695.1 | *Cinara cuneomaculata* | BCc4 | 430,960 | 368 | 23.60 | PRJNA224116 | SAMEA5379123 |
| G42 | NZ_LR217707.1 | *Cinara kochiana kochiana* | BCkk | 433,740 | 374 | 23.30 | PRJNA224116 | SAMEA5379131 |
| G43 | NZ_LR217717.1 | *Cinara laricifoliae* | BCl | 436,713 | 374 | 22.30 | PRJNA224116 | SAMEA5379133 |
| G44 | NZ_LR217732.1 | *Cinara pseudotaxifoliae* | BCp2 | 446,658 | 374 | 24.1 | PRJNA224116 | SAMEA5379137 |
| G45 | NZ_LR217692.1 | *Cinara cf. splendens/pseudotsugae* | BCs/p | 444,797 | 378 | 23.90 | PRJNA224116 | SAMEA5379121 |
| G46 | NZ_LR217722.1 | *Cinara splendens* | BCs1 | 445,237 | 377 | 23.60 | PRJNA224116 | SAMEA5379141 |
| G47 | NZ_LT667500.1 | *Cinara fornacula* | BCf | 447,673 | 375 | 28.10 | PRJNA224116 | SAMEA4556317 |
| G48 | NZ_LR025085.1 | *Cinara strobi* | BCs2 | 440,140 | 372 | 23.90 | PRJNA224116 | SAMEA4598800 |
| G49 | CP001817.1 | *Cinara tujafilina* [37] | BCt | 444,925 | 395 | 23.00 | PRJNA41379 | SAMN02603413 |
| G50 | NZ_LT667503.1 | *Cinara confinis* | BCc5 | 443,747 | 384 | 24.00 | PRJNA224116 | SAMEA4556315 |
| G51 | NZ_LN890285.1 | *Tuberolachnus salignus* | BTs | 421,426 | 377 | 21.60 | PRJNA224116 | SAMEA3598223 |
| G52 | NZ_CP032996.1 | *Therioaphis trifolii* [38] | BTt | 419,293 | 390 | 20.20 | PRJNA224116 | SAMN09980522 |
| G53 | NZ_CP032999.1 | *Sarucallis kahawaluokalani* [38] | BSk | 428,356 | 390 | 24.80 | PRJNA224116 | SAMN09980519 |
| G54 | NZ_CP032998.1 | *Stegophylla sp.* [38] | BSs | 412,404 | 353 | 23.00 | PRJNA224116 | SAMN09980520 |
| G55 | NZ_CP033012.1 | *Anoecia oenotherae* [38] | BAo | 548,691 | 444 | 22.70 | PRJNA224116 | SAMN09980506 |
| G56 | NZ_CP034852.1 | *Thelaxes californica* [38] | BTc | 522,699 | 453 | 22.60 | PRJNA224116 | SAMN09980521 |
| G57 | NZ_AP019379.1 | *Nipponaphis monzeni* [39] | BNm | 587,781 | 445 | 22.30 | PRJNA224116 | SAMD00154410 |
|  | NZ_AP010872.1 | *Candidatus Ishikawaella capsulata* [67] |  | 745,590 | 620 | 30.20 | PRJNA224116 | SAMD00061091 |
|  | NC_000913.3 | *Escherichia coli* [110] |  | 4,641,652 | 4,315 | 50.80 | PRJNA57779 | SAMN02604091 |
|  | NZ_CP014768.1 | *Shigella* sp. |  | 4,558,287 | 4,270 | 50.80 | PRJNA224116 | SAMN04548868 |

Notes: Aphid Host with [] indicates the source of this genome sequence, Code with “^1-5^” are used to differentiate *Buchnera* with the same code.

**Supplementary References**

1. Cassone BJ, Wenger JA, Michel PA. Whole genome sequence of the soybean aphid endosymbiont *Buchnera aphidicola* and genetic differentiation among biotype-specific strains. J Genomics. 2015;3:85-94. doi: 10.7150/jgen.12975.
2. van Ham R, Kamerbeek J, Palacios C, Rausell C, Abascal F, Bastolla U, Fernández JM, Jiménez L, Postigo M, Silva FJ, et al. Reductive genome evolution in *Buchnera aphidicola*. Proc Natl Acad Sci U S A. 2003;100:581-6. doi: 10.1073/pnas.0235981100.
3. Pérez-Brocal V, Gil R, Ramos S, Lamelas A, Postigo M, Michelena JM, Silva FJ, Moya A, Latorre A. A small microbial genome: the end of a long symbiotic relationship? Science. 2006;314:312-3. doi: 10.1126/science.1130441.
4. Lamelas A, Gosalbes MJ, Moya A, Latorre A. New clues about the evolutionary history of metabolic losses in bacterial endosymbionts, provided by the genome of *Buchnera aphidicola* from the aphid *Cinara tujafilina*. Appl Environ Microbiol. 2011;77:4446-54. doi: 10.1128/AEM.00141-11
5. Chong RA, Park H, Moran NA. Genome evolution of the obligate endosymbiont *Buchnera aphidicola*. Mol Biol Evol. 2019;36:1481-9. doi: 10.1093/molbev/msz082.
6. Kutsukake M, Moriyama M, Shigenobu S, Meng XY, Nikoh N, Noda C, Kobayashi S, Fukatsu K. Exaggeration and cooption of innate immunity forsocial defense. Proc Natl Acad Sci U S A. 2019;116:8950-9. doi: 10.1073/pnas.1900917116.
7. Monnin D, Jackson R, Kiers ET, Bunker M, Ellers J, Henry LM. Parallel evolution in the integration of a co-obligate aphid symbiosis. Curr Biol. 2020;30:1949-57. doi: 10.1016/j.cub.2020.03.011.
8. Zhang Y, Su X, Harris AJ, Caraballo-Ortiz MA, Ren ZM, Zhong Y. Genetic structure of the bacterial endosymbiont *Buchnera aphidicola* from its host aphid *Schlechtendalia chinensis* and evolutionary implications. Curr Microbiol. 2018;75:309-15. doi: 10.1007/s00284-017-1381-0.
9. Zhang B, Leonard SP, Li YY, Moran NA. Obligate bacterial endosymbionts limit thermal tolerance of insect host species. Proc Natl Acad Sci U S A. 2019;116:24712-8. doi: 10.1073/pnas.1915307116.
10. Nikoh N, Hosokawa T, Oshima K, Hattori M, Fukatsu T. Reductive evolution of bacterial genome in insect gut environment. Genome Biol Evol. 2011;3:702-14. doi: 10.1093/gbe/evr064.
11. Chen WB, Shakir S, Bigham M, Richter A, Fei ZJ, Jander G. Genome sequence of the corn leaf aphid (*Rhopalosiphum maidis* Fitch). Gigascience. 2019;8:giz033. doi: 10.1093/gigascience/giz033.
12. Mathers TC, Mugford ST, Hogenhout SA, Tripathi L. Genome sequence of the banana aphid, *Pentalonia nigronervosa* Coquerel (Hemiptera: Aphididae) and its symbionts. G3 (Bethesda). 2020;10:4315-21. doi: 10.1534/g3.120.401358.
13. Jiang Z, Jones DH, Khuri S, Tsinoremas NF, Wyss T, Jander G, Wilson AC. Comparative analysis of genome sequences from four strains of the *Buchnera aphidicola* Mp endosymbion of the green peach aphid, *Myzus persicae*. BMC Genomics. 2013;14:917. doi: 10.1186/1471-2164-14-917.
14. Moran NA, McLaughlin HJ, Sorek R. The dynamics and time scale of ongoing genomic erosion in symbiotic bacteria. Science. 2009;323:379-82. doi: 10.1126/science.1167140.
15. MacDonald SJ, Thomas GH, Douglas AE. Genetic and metabolic determinants of nutritional phenotype in an insect-bacterial symbiosis. Mol Ecol. 2011;20:2073-84. doi: 10.1111/j.1365-294X.2011.05031.x.
16. Degnan PH, Ochman H, Moran NA. Sequence conservation and functional constraint on intergenic spacers in reduced genomes of the obligate symbiont *Buchnera*. PLoS Genet. 2011;7:e1002252. doi: 10.1371/journal.pgen.1002252.
17. Riley M, Abe T, Arnaud MB, Berlyn MK, Blattner FR, Chaudhuri RR, Glasner JD, Horiuchi T, Keseler IM, Kosuge T, et al. *Escherichia coli* K-12: a cooperatively developed annotation snapshot--2005. Nucleic Acids Res. 2006;34:1-9. doi: 10.1093/nar/gkj405.
