# Supplementary material for: Comparative genomics of the primary endosymbiont Buchnera aphidicola in aphid hosts and their coevolutionary relationships
Source: BMC Biol. 2024 Jun 20;22:137. doi: 10.1186/s12915-024-01934-w (PMC11188193; doi:10.1186/s12915-024-01934-w)
Supplement: Supplementary file 2 — Additional file 2: Figures S1-S3. Fig. S1. The BI tree of aphids without partitioning. Stars at the branches represent bootstrap support of BI 1.00. The colors correspond to the subfamilies of host aphids represented, Blue: Aphidinae, Purple: Eriosomatinae, Green: Lachninae. Fig. S2. The ML tree of aphids with partitioning. Stars at the branches represent bootstrap support of ML 100%. The colors correspond to the subfamilies of host aphids represented, Blue: Aphidinae, Purple: Eriosomatinae, Green: Lachninae. Fig. S3. The ML tree of aphids without partitioning. Stars at the branches represent bootstrap support of ML 100%. The colors correspond to the subfamilies of host aphids represented, Blue: Aphidinae, Purple: Eriosomatinae, Green: Lachninae. [file 12915_2024_1934_MOESM2_ESM.docx]

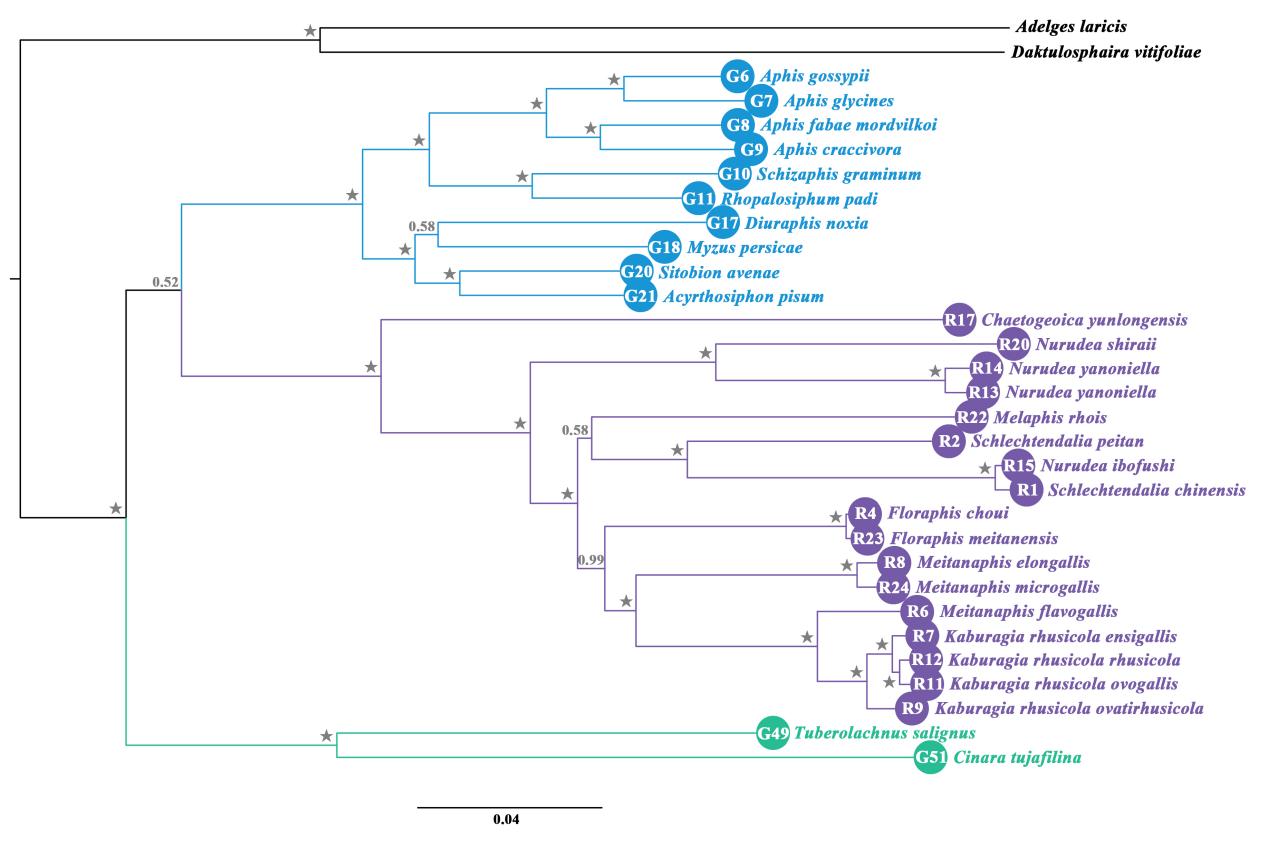


**Figure S1** The BI tree of aphids without partitioning. Stars at the branches represent bootstrap support of BI 1.00. The colors correspond to the subfamilies of host aphids represented, Blue: Aphidinae, Purple: Eriosomatinae, Green: Lachninae.


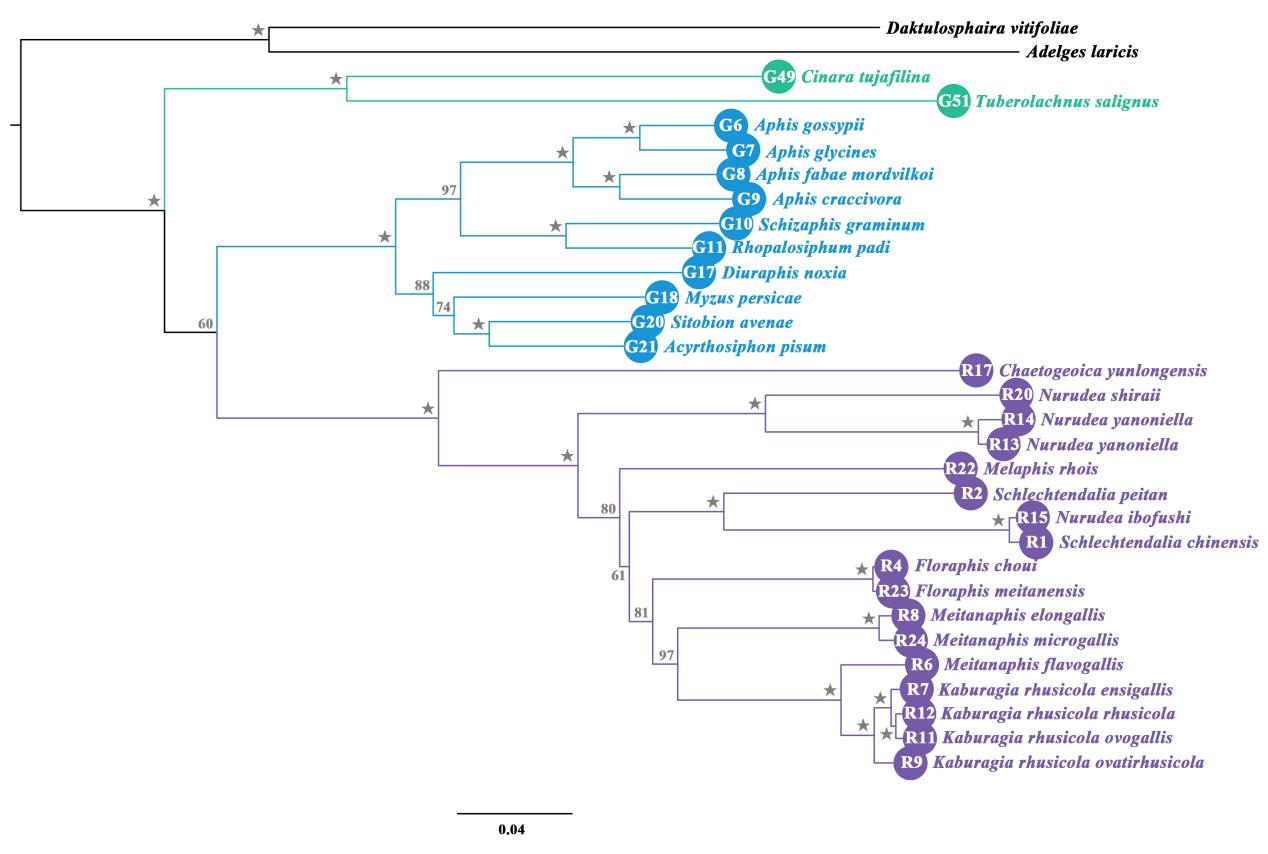


**Figure S2** The ML tree of aphids with partitioning. Stars at the branches represent bootstrap support of ML 100%. The colors correspond to the subfamilies of host aphids represented, Blue: Aphidinae, Purple: Eriosomatinae, Green: Lachninae.


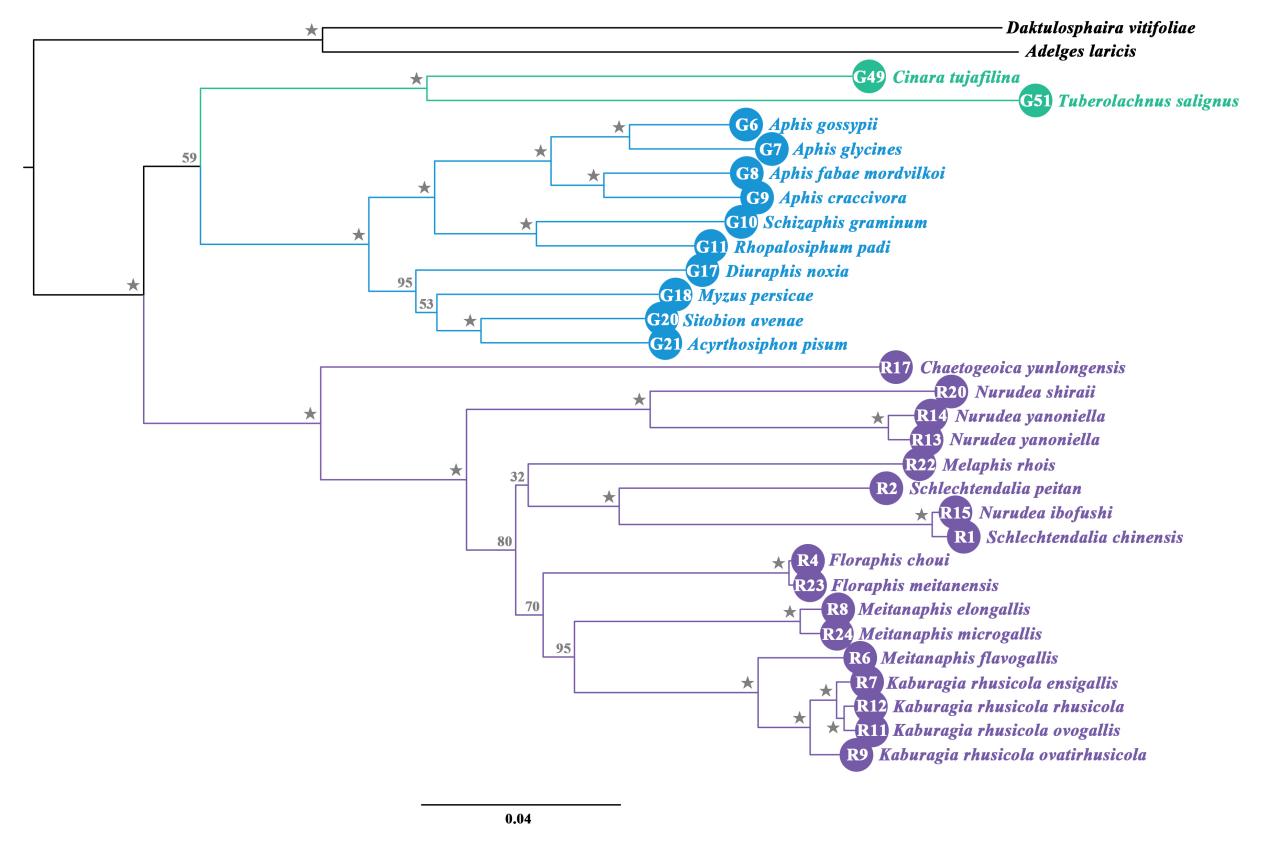


**Figure S3** The ML tree of aphids with partitioning. Stars at the branches represent bootstrap support of ML 100%. The colors correspond to the subfamilies of host aphids represented, Blue: Aphidinae, Purple: Eriosomatinae, Green: Lachninae.
